# Hemodynamic Impact of Cipepofol vs Propofol During Anesthesia Induction in Patients With Severe Aortic Stenosis: A Randomized Clinical Trial

**DOI:** 10.1001/jamasurg.2025.1299

**Published:** 2025-05-21

**Authors:** Tingting Ni, Xiaoxia Zhou, Shuguang Wu, Tao Lv, Yujiao Hu, Qi Gao, Ge Luo, Chen Xie, Jingcheng Zou, Yuexiu Chen, Linqian Zhao, Jie Xiao, Xincheng Tao, Yu Yi, Zhili Xu, Tingting Wang, Junyu Zhou, Yuanyuan Yao, Min Yan

**Affiliations:** 1 Department of Anesthesiology, The Second Affiliated Hospital of Zhejiang University School of Medicine, Hangzhou, China; 2 Department of Anesthesiology, The Fourth Affiliated Hospital of Zhejiang University School of Medicine, Yiwu City, China

## Abstract

**Importance:**

Postinduction hemodynamic instability is a frequent complication among patients with severe aortic stenosis (AS). Using cipepofol as the anesthesia agent may reduce the incidence and severity of hemodynamic instability.

**Objective:**

To assess whether cipepofol outperforms propofol in maintaining postinduction hemodynamic stability in patients with AS.

**Design, Setting, and Participants:**

This single-center, randomized clinical trial was conducted from June 29, 2023, to July 8, 2024, at the Second Affiliated Hospital of Zhejiang University School of Medicine in China. Patients with AS scheduled for transcatheter aortic valve replacement (TAVR) were eligible for inclusion.

**Interventions:**

Participants were randomized 1:1 to receive either cipepofol or propofol as anesthesia induction agents at equipotent doses.

**Main Outcomes and Measures:**

The primary outcome was the area under the curve (AUC) of the mean arterial pressure (MAP) difference from baseline during the initial 15 minutes postinduction.

**Results:**

A total of 124 patients with AS scheduled for TAVR were randomized into either the cipepofol group (n = 62) or the propofol group (n = 62). Of 124 patients randomized, 1 patient from each group was excluded due to ineligibility for the TAVR procedure, and data were analyzed for 122 patients (61 patients per group) based on the intention-to-treat principle. Among 122 total patients, mean (SD) age was 72.2 (5.0) years, and 53 patients (43.4%) were female. The cipepofol group exhibited a significantly smaller median (IQR) AUC (−8505.0 mm Hg · s [−12 402.8 to −5130.0]) compared with the propofol group (−13 189.0 mm Hg · s [−17 006.7 to −7593.3]; *P* < .001). Moreover, compared with the propofol group, the cipepofol group demonstrated a significantly lower incidence of postinduction hypotension (70.5% vs 88.5%; *P* = .01) and required a smaller median (IQR) dose of norepinephrine during the first 15 minutes postinduction (6.0 μg [0.0-10.0] vs 10.0 μg [5.0-20.0]; *P* = .006). Additionally, the 2 groups’ bispectral indices were comparable.

**Conclusions and Relevance:**

In this randomized clinical trial, cipepofol provided superior hemodynamic stability as an induction agent compared to propofol at equipotent doses and similar anesthesia depths for patients with AS. Therefore, cipepofol could serve as an alternative induction agent to propofol for patients at high cardiovascular risk.

**Trial Registration:**

ClinicalTrials.gov Identifier: NCT05881291

## Key Points

**Question:**

Does cipepofol offer superior hemodynamic stability compared to propofol as an anesthesia induction agent?

**Findings:**

In this randomized clinical trial involving 122 adults with aortic stenosis with anesthesia induced with either cipepofol or propofol, the cipepofol group exhibited a significantly smaller area under the curve for changes in mean arterial pressure relative to baseline compared to the propofol group.

**Meaning:**

Cipepofol may be a safer alternative induction anesthetic for patients with severe aortic stenosis.

## Teaser

This randomized clinical trial conducted in China assesses whether cipepofol outperforms propofol in maintaining postinduction hemodynamic stability in patients with severe aortic stenosis.

## Introduction

Transcatheter aortic valve replacement (TAVR) is a less invasive alternative to surgical aortic valve replacement for patients with severe aortic stenosis (AS).^1^ Arterial hypotension is a common complication that can occur from anesthesia induction to the first surgical incision,^2,3,4,5^ particularly in older patients with compromised preoperative physical status.^3,5,6^ AS is characterized by left ventricular outflow obstruction, leading to inadequate cardiac output and heart failure.^7^ A significant proportion of patients (24%-45%) have concomitant coronary artery disease.^8^ Peripheral vascular resistance is crucial for maintaining blood pressure in this population. However, during the induction of general anesthesia, anesthetic-induced vasodilation and bradycardia can cause refractory hypotension, which is life threatening.^9^ Therefore, preventing severe hypotension during anesthesia induction is critical for patient safety.

Propofol is a commonly used induction anesthetic for patients undergoing TAVR; however, its use is linked to excessive vasodilation and decreased cardiac output, frequently leading to arterial hypotension.^10,11^ Therefore, there is an urgent need to identify an alternative anesthetic that offers a similar anesthetic efficacy to propofol while offering better hemodynamic stability. Cipepofol (also known as ciprofol) is a short-acting propofol analog and potentiator of the γ-aminobutyric acid type A receptor. The addition of a cyclopropylethyl group to cipepofol’s core phenol structure results in a higher receptor affinity than that of propofol.^12,13^ Cipepofol has demonstrated rapid anesthesia onset, effective sedation at lower doses, and low injection site pain.^12,13,14,15,16^ Moreover, its efficacy and safety as a general anesthetic, particularly in older patients, make it a reliable choice for anesthesia induction in this population.^17^ Although several randomized clinical trials have assessed the hemodynamic effects of cipepofol during anesthesia,^18,19,20,21,22^ none have specifically investigated its use for inducing general anesthesia in patients with severe AS.

In this study, we sought to assess whether cipepofol outperforms propofol in maintaining hemodynamic stability in patients with AS during the first 15 minutes after induction.^13^ Hemodynamic changes were closely monitored for 15 minutes because severe hypotension and hemodynamic instability commonly occur during this period after anesthesia induction. After this initial period, hemodynamics gradually stabilize as anesthetics distribute throughout the body and their pharmacodynamic effects decline.^23^

It was hypothesized that cipepofol would better mitigate the risk of postinduction hemodynamic instability than propofol at equipotent doses. The hypothesis was tested by determining and comparing the area under the curve (AUC) of the mean arterial pressure (MAP) difference from baseline within the first 15 minutes postinduction of general anesthesia in patients with AS between the 2 compounds.

## Methods

### Study Design

This single-site randomized clinical trial was conducted from June 29, 2023, to July 8, 2024, in adherence with the Consolidated Standards of Reporting Trials (CONSORT) reporting guidelines. The protocol received ethics approval from the Second Affiliated Hospital of Zhejiang University School of Medicine in China on April 12, 2023. Written informed consent was obtained from all participants before enrollment. The trial was prospectively registered on ClinicalTrials.gov (NCT05881291) under the primary investigator, Dr Yan. The trial protocol is available in Supplement 1.

### Study Population

Patients were eligible if they met all the following inclusion criteria: (1) scheduled for elective TAVR; (2) expected surgery duration of 1 or more hours to 3 or fewer hours; (3) planned use of general anesthesia with endotracheal intubation; (4) age between 60 and 85 years; (5) body mass index of 18 to 30 (calculated as weight in kilograms divided by height in meters squared) at screening; (6) American Society of Anesthesiologists physical status scale III to IV; and (7) capable of understanding the study and providing informed consent.

Exclusion criteria were the following: (1) preoperative tracheal intubation; (2) known allergies to eggs, soy products, opioids, their reversal agents, or propofol; (3) shock or hypotension unresponsive to vasopressors; (4) history of neurological or psychiatric disorders; (5) hemoglobin less than 10 g/dL (to convert hemoglobin from grams per deciliter to grams per liter, multiply by 10) at screening; and (6) long-term use of analgesics or sedatives. Patients unwilling to participate before anesthesia induction were withdrawn.

### Data Collection

The MAP was continuously recorded and averaged every 30 seconds by a DoCare information system (Medicalsystem Co). An in-room observer recorded the number of boluses and the total amount of vasoactive drugs administered in the first 15 minutes after anesthesia induction. Other data describing patient characteristics, surgical procedures, and anesthetics were extracted from the electronic anesthesia records or the patient’s clinical notes.

### Randomization and Blinding

Randomization followed a 1:1 ratio using a computer-generated table (SPSS [IBM]) prepared before the study, with assignments concealed in sequentially numbered opaque envelopes managed by an independent investigator. On surgery day, anesthesiologists received the envelopes per the randomization schedule. In this single-blind study, patients were blinded to treatment allocation, while anesthesiologists could not be blinded due to medication management requirements. The outcome assessors, data analysts, and in-room observers were blinded to group assignments, and anesthesiologists administering drugs were not involved in outcome assessment.

### Standardized Surgical Procedure

TAVR is recommended in older patients (≥75 years) or in those who are considered high risk (Society of Thoracic Surgeons Predicted Risk of Mortality score or EuroSCORE II >8%) or unsuitable for surgery according to the 2021 European Society of Cardiology (ESC)/European Association for Cardio-Thoracic Surgery (EACTS) guidelines for the management of valvular heart disease.^24^ Comprehensive monitoring was initiated upon entering the operating room, with radial artery catheterization, central venous access, and, for CoreValve (Medtronic) patients, a temporary pacemaker via the internal jugular vein to mitigate heart block risk. In the propofol group, patients received propofol (Fresenius Kabi Deutschland GmbH), 1 mg/kg, over 30 seconds until loss of consciousness (LOC), defined as the disappearance of the eyelash reflex, occurred. If LOC was not achieved within 1 minute after the initial administration of propofol, a top-up dose of 50% of the initial dose was administered, followed by another top-up dose if LOC had not occurred within 2 minutes. Subsequently, alfentanil, 20 to 25 μg/kg, and rocuronium, 0.6 mg/kg, were given, with tracheal intubation performed 2 minutes later. Sedation depth was adjusted to maintain a bispectral index (BIS) between 40 and 60 by titrating the propofol infusion rate. In the cipepofol group (Shenyang Haisco Pharmaceutical Co), induction used cipepofol, 0.2 mg/kg, with dosage adjustments made to achieve the same BIS target as in the propofol group (detailed in eMethods 1 in Supplement 2). A meta-analysis demonstrated that cipepofol exhibited 4- to 5-fold greater potency than propofol for anesthesia.^25^ The remaining induction protocol mirrored that of the propofol group. Propofol or cipepofol infusion was discontinued after surgery, and residual muscle paralysis was reversed with sugammadex or a combination of atropine and neostigmine to ensure a train of four (TOF) ratio of 0.9 or higher.

The endotracheal tube was removed once the patient had regained consciousness and demonstrated spontaneous and stable respiration, stable hemodynamics, and no signs of bleeding. Postinduction hypotension (PIH), defined as a MAP reduction of more than 20% from baseline or a MAP less than 65 mm Hg for at least 1 minute. Any MAP drop by more than 20% from baseline or a MAP less than 65 mm Hg for at least 1 minute within the first 15 minutes after general anesthesia induction triggered the administration of norepinephrine boluses (5 μg). If hypotension persisted after 2 boluses, a norepinephrine infusion (0.02-0.3 μg/kg/min) was initiated and continued until the MAP became stable and reached 65 mm Hg or higher. If hypotension reoccurred, 2 boluses of norepinephrine were repeated. The baseline MAP was defined as the mean MAP during the 5 minutes preceding induction.

### Study Outcomes

#### Primary and Secondary Outcomes

The primary outcome measure was the AUC during the initial 15 minutes postinduction. Secondary outcomes included the incidence of hypotension, need for temporary pacemaker activation (heart rate <50 bpm), lowest MAP, cumulative vasopressor dose, anesthetic dosage, BIS index within 15 minutes postinduction, time to LOC, percentage of time with a BIS value of 40 to 60, and postoperative recovery indicators (extubation time, postanesthesia care unit (PACU) stay duration, hospital stay length, and Quality of Recovery-15 (QoR-15) score (see eTable 3 in Supplement 2 for details on the QoR-15 score). Extubation duration refers to the time interval from the cessation of anesthetic agents to the removal of the endotracheal tube.

#### Safety Outcomes

Safety was evaluated in terms of the frequency of adverse events, alterations in vital signs, postoperative recovery, and injection site pain, which was assessed with a 4-point scale (eTable 4 in Supplement 2).

### Sample Size Calculation

The primary objective of this study was to measure the AUC within 15 minutes postinduction of general anesthesia. Our pilot study showed a mean (SD) AUC of −10 706.4 (6877.0) mmHg · s for propofol. Experts^26^ consider a 33% AUC difference within this time frame to be clinically relevant. A total of 124 participants (62 per treatment group) provided 80% power to detect differences between propofol and cipepofol at a 2-sided α of .05, accounting for 5% attrition.

### Statistical Analysis

This study followed the intention-to-treat principle, including all patients randomized to cipepofol or propofol groups, with only preconversion data used for those requiring open-chest surgery to maintain randomization integrity and reduce selection bias. For the primary outcome measure, the Wilcoxon rank-sum test was used to compare the AUC within the first 15 minutes after anesthesia induction between groups. The AUC below the baseline MAP was calculated as

 ∑ (((S_i_ − S_baseline_) + (S_i_ _−_ _1_ − S_baseline_))/2 × ΔX),

where S_baseline_ is the baseline MAP, S_i_ is the MAP at minute i (i = 1…15), and ΔX is the time interval between measurements.

We used the log-rank test to compare group differences in time to extubation, PACU stay, and hospital stay, accounting for censored data. Additionally, data distributions were assessed with the Shapiro-Wilk test; continuous variables were analyzed using *t* tests or Mann-Whitney *U* tests, and binary variables were analyzed with χ^2^ or Fisher exact tests, with significance at *P* < .05. We used SAS Enterprise Guide version 8.3 (SAS Institute) for analyses and GraphPad Prism version 9.1.0 (GraphPad Software) for visualizations (detailed parameters are provided in the eMethods 2 in Supplement 2).

## Results

### Baseline Characteristics

**Figure 1.**
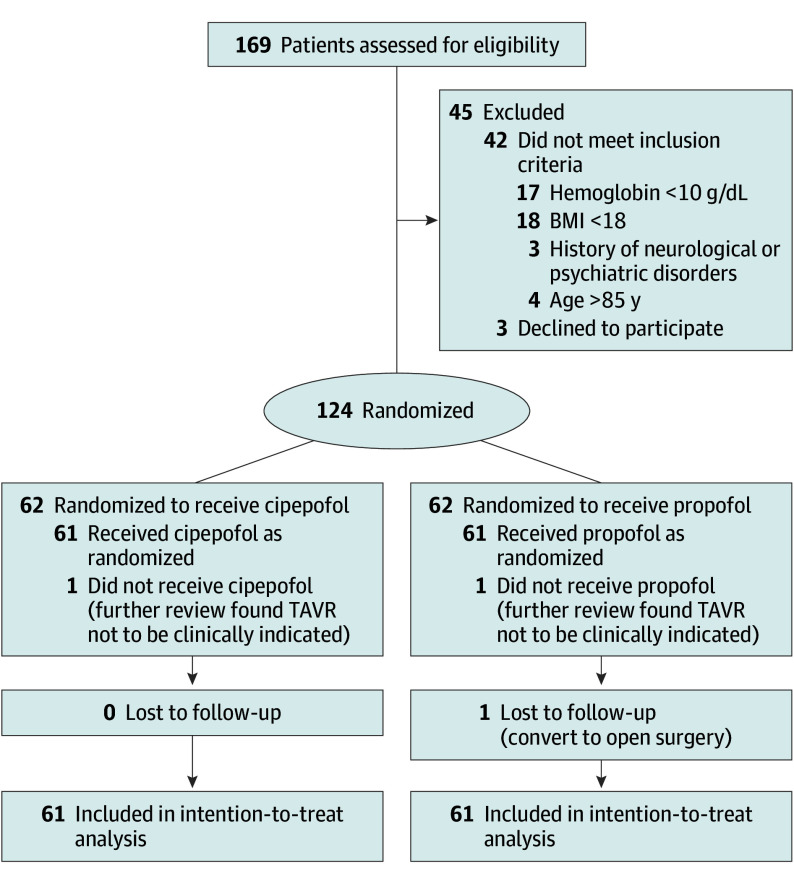
CONSORT Diagram BMI indicates body mass index (calculated as weight in kilograms divided by height in meters squared); TAVR, transcatheter aortic valve replacement. SI conversion factor: To convert hemoglobin from g/dL to g/L, multiply by 10.

A total of 169 patients were initially screened, 45 of whom were excluded (42 did not meet the inclusion criteria, and 3 declined to participate). Finally, 124 patients were included and randomized to the cipepofol (n = 62) and propofol (n = 62) groups. One patient was excluded from each group after randomization due to inappropriateness for the TAVR procedure. Additionally, 1 patient in the propofol group was converted to open-chest surgery. Following the intention-to-treat principle, the data were analyzed up to the conversion point, leading to a full analysis set of 122 individuals (61 in each group) (Figure 1).

Among 122 total patients, mean (SD) age was 72.2 (5.0) years, and 53 patients (43.4%) were female. There were no significant differences between the 2 groups in terms of demographic variables or comorbidities (Table 1).

**Table 1.** Baseline Demographic and Intraoperative Characteristics of the Patients

Variable	No. (%)	
	Cipepofol (n = 61)	Propofol (n = 61)
Age, mean (SD), y	71.9 (4.5)	72.4 (5.5)
Sex		
Female	29 (47.5)	24 (39.3)
Male	32 (52.5)	37 (60.7)
BMI, mean (SD)^a^	23.1 (3.4)	22.9 (2.5)
Smoking	11 (18.0)	8 (13.1)
Drinking alcohol	8 (13.1)	9 (14.8)
NYHA functional class		
II	32 (52.5)	30 (49.2)
III	25 (41.0)	29 (47.5)
IV	4 (6.6)	2 (3.3)
STS-PROM score, median (IQR), %	2.4 (1.6-4.0)	2.6 (1.6-4.2)
Comorbidities		
Hypertension	44 (72.1)	38 (62.3)
Diabetes	12 (19.7)	13 (21.3)
Coronary heart disease	13 (21.3)	16 (26.2)
Atrial fibrillation	6 (9.8)	10 (16.4)
COPD	12 (19.7)	5 (8.2)
Prior MI	1 (1.6)	1 (1.6)
Prior stroke	9 (14.8)	3 (4.9)
Prior PCI	9 (14.8)	7 (11.5)
Prior pacemaker implantation	2 (3.3)	1 (1.6)
Prior pulmonary infection	0	2 (3.3)
Only AS	29 (47.5)	30 (49.2)
Only AR	14 (23.0)	15 (24.6)
AS and AR	18 (29.5)	16 (26.2)
Medication on admission		
β-Adrenergic receptor blocker	13 (21.3)	10 (16.4)
ACEIs/ARBs	14 (23.0)	19 (31.1)
Antiplatelet	27 (44.3)	20 (32.8)
Anticoagulation	2 (3.28)	3 (4.92)
Lipid-lowering drug	24 (39.3)	20 (32.8)
Echocardiographic parameters		
EF, median (SD), %	59.7 (10.4)	59.5 (10.2)
Mean gradient, median (IQR), mm Hg	41.0 (18.0-49.0)	42.0 (30.0-57.0)
Maximum velocity, median (IQR), m/s	4.2 (2.8-4.6)	4.2 (3.5-4.8)
AVA, median (IQR), cm^2^	0.8 (0.7-1.1)	0.8 (0.6-1.0)
Laboratory data		
Hemoglobin, mean (SD), g/dL	12.9 (1.6)	12.8 (1.5)
BUN, mean (SD), mg/dL	18.49 (6.44)	18.49 (6.72)
Serum creatinine, median (IQR), mg/dL	0.80 (0.68-1.02)	0.84 (0.75-1.00)
BNP, median (IQR), pg/mL	194.4 (78.7-494.6)	213.6 (72.3-344.1)
CK-MB, median (IQR), U/L	11.0 (9.0-16.0)	10.0 (8.0-13.0)
cTn, median (IQR), ng/mL	0.01 (0.01-0.03)	0.02 (0.01-0.03)
Physiological monitoring parameters		
Preoperative HR, median (IQR), beats/min	76 (66-81)	73 (69-82)
Preoperative MAP, mean (SD), mm Hg	97 (11)	97 (13)
Baseline BIS, median (IQR), %	98 (97-98)	98 (97-98)
Procedure data		
Duration of surgery, median (IQR), min	85 (70-100)	80 (65-105)
Duration of anesthesia, median (IQR), min	115 (99-138)	106 (92-136)
Fluid volume, median (IQR), mL	1500 (1000-1500)	1500 (1000-1500)
Blood loss, median (IQR), mL	50 (30-50)	50 (30-50)
Urine, median (IQR), mL	300 (200-500)	250 (150-460)
Contrast, median (IQR), mL	100 (80-129)	100 (80-120)
Valve type		
Balloon expandable	19 (31.2)	19 (31.7)^b^
Self-expanding	42 (68.9)	41 (68.3)^b^

Abbreviations: ACEI; angiotensin-converting enzyme inhibitor; AR, aortic regurgitation; ARB, angiotensin receptor blocker; AS, aortic stenosis; AVA, aortic valve area; BIS, bispectral index; BMI, body mass index; BNP, B-type natriuretic peptide; BUN, blood urea nitrogen; CK-MB, creatine kinase–MB fraction; COPD, chronic obstructive pulmonary disease; cTn, cardiac troponin T; EF, ejection fraction; HR, heart rate; MAP, mean arterial pressure; MI, myocardial infarction; NYHA, New York Heart Association; PCI, percutaneous coronary intervention; STS-PROM, Society of Thoracic Surgeons Predicted Risk of Mortality.

SI conversion factors: To convert hemoglobin from g/dL to g/L, multiply by 10; blood urea nitrogen, from mg/dL to mmol/L, multiply by 0.357; serum creatinine, from mg/dL to μmol/L, multiply by 88.4; BNP, from ng/L to pg/mL, multiply by 1, cTn, from ng/mL to μg/L, multiply by 1.

^a^ Calculated as weight in kilograms divided by height in meters squared.

^b^ One participant in the propofol group required conversion to open-heart surgery, resulting in missing data on the valve type.

### Primary Outcome

**Figure 2.**
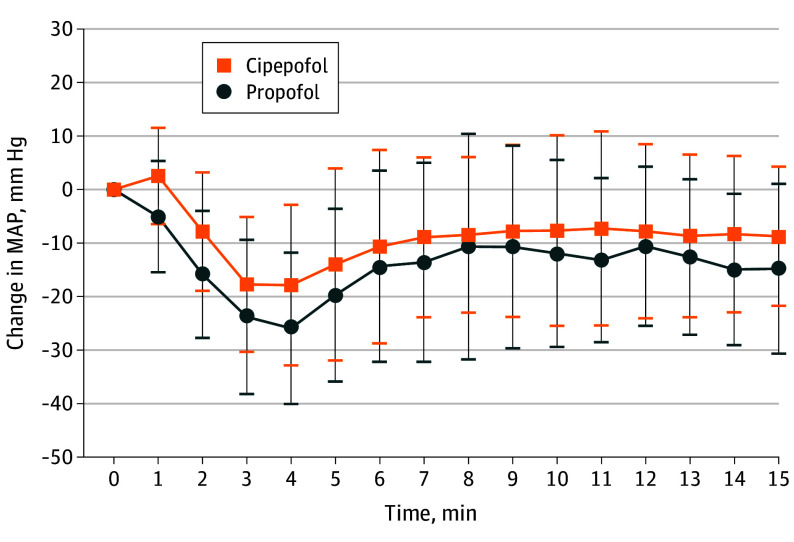
Changes in Mean Arterial Pressure (MAP) During the First 15 Minutes Postinduction for Cipepofol and Propofol Groups

Compared with the propofol group, the cipepofol group exhibited a significantly smaller median (IQR) AUC (−8505.0 mm Hg · s [−12 402.8 to −5130.0] vs −13 189.0 mm Hg · s [−17 006.7 to −7593.3]; *P* < .001), representing a 35% reduction in AUC (Table 2). Changes in MAP postadministration are shown in Figure 2. Results for the per-protocol set (as shown in eTable 2 in Supplement 2) were consistent with the full analysis set.

**Table 2.** Comparison of Hemodynamic Effects Between Cipepofol and Propofol in Patients Undergoing Induction of Anesthesia

Variable	Median (IQR)		*P* value
	Cipepofol (n = 61)	Propofol (n = 61)	
Primary outcome			
AUC of MAP difference from baseline, mmHg · s	−8505.0 (−12 402.8 to −5130.0)	−13 189.0 (−17 006.7 to −7593.3)	<.001
Secondary outcomes			
Postinduction hypotension, No. (%)	43 (70.5)	54 (88.5)	.01
Lowest MAP, mean (SD), mm Hg	66 (10)	62 (11)	.007
Pacemaker activation, No. (%)	5 (8.2)	8 (13.1)	.38
Vasopressor use, μg			
Norepinephrine dose within 15 min of induction	6.0 (0.0 to 10.0)	10.0 (5.0 to 20.0)	.006
Norepinephrine dose during the entire procedure	356.0 (156.0 to 553.0)	443.0 (264.0 to 724.0)	.04
Epinephrine dose during the entire procedure	14.0 (0.0 to 67.0)	20.0 (0.0 to 50.0)	.68
Time to LOC, s	56 (50 to 63)	55 (49 to 60)	.55
Dose of cipepofol or propofol, mg			
For induction^a^	12.0 (10.0 to 14.2)	60.0 (50.0 to 70.0)	<.001
Total amount of drug administered^a^	120.0 (102.5 to 152.5)	538.0 (420.0 to 610.0)	<.001
Top-up dose during induction, No. (%)	7 (11.5)	9 (14.8)	.59
Dose of alfentanil, μg			
For induction	1200.0 (1000.0 to 1400.0)	1200.0 (1000.0 to 1400.0)	.96
Total amount of drug administered	2800.0 (2520.0 to 3200.0)	2700.0 (2300.0 to 3060.0)	.29
Dose of rocuronium, mg			
For induction	35.0 (30.0 to 42.0)	36.0 (33.0 to 41.0)	.57
Total amount of drug administered	39.0 (34.0 to 45.0)	40.0 (34.0 to 47.0)	.53
% of Time with BIS value 40-60, %	73.5 (54.3 to 82.2)	63.0 (44.7 to 76.0)	.06
Extubation duration, min	35 (25 to 45)	30 (20 to 40)	.13
Length of PACU stay, min	75 (65 to 85)	70 (62 to 80)	.59
Length of hospital stay, d	8.0 (7.0 to 9.0)	9.0 (9.0 to 10.0)	.35
QoR-15 score, mean (SD)	125.7 (9.4)	122.2 (12.2)	.08

Abbreviations: AUC, area under the curve; BIS, bispectral index; LOC, loss of consciousness; MAP, mean arterial pressure; PACU, postanesthesia care unit; QoR-15, Quality of Recovery-15.

^a^ Cipepofol doses were converted to propofol equivalents (conversion factor 4-5). Median cipepofol induction dose (12.0 mg) corresponds to 48.0-60.0 mg propofol equivalent, consistent with observed propofol dosing (60.0 mg). Total cipepofol (120.0 mg) equals 480.0-600.0 mg propofol equivalent, aligning with total propofol usage (538.0 mg).

### Secondary Outcomes

Following the primary outcome analysis of AUC, further analyses assessed additional indicators of hemodynamic instability between the 2 groups, such as PIH and the use of vasopressors. PIH occurred in 43 patients in the cipepofol group (70.5%), whereas in the propofol group, it occurred in 54 patients (88.5%) (*P* = .01) (Table 2). The lowest mean (SD) MAPs in the cipepofol and propofol groups were 66 (10) mm Hg and 62 (11) mm Hg, respectively, and the difference was significant (*P* = .007). Pacemaker activation occurred for 5 patients in the cipepofol group (8.2%) vs 8 patients in the propofol group (13.1%) (*P* = .38).

Median (IQR) norepinephrine dosages were significantly lower in the cipepofol group than in the propofol group both within the first 15 minutes postinduction (6.0 μg [0.0-10.0] vs 10.0 μg [5.0-20.0]; *P* = .006) (eFigure 1 in Supplement 2) and over the entire procedure (356.0 μg [156.0-553.0] vs 443.0 μg [264.0-724.0]; *P* = .04), while epinephrine doses did not significantly differ (14.0 μg [0.0-67.0] vs 20.0 μg [0.0-50.0]; *P* = .68). The median (IQR) time to LOC was also comparable: 56 seconds (50-63) for the cipepofol group vs 55 seconds (49-60) for the propofol group (*P* = .55) (Table 2).

The induction success rates were 100% in both groups. Top-up doses were needed for 7 patients in the cipepofol group (11.5%) and 9 patients in the propofol group (14.8%) (*P* = .59). Additionally, there were no statistically significant differences in the doses of alfentanil and rocuronium used between the 2 groups during either the induction phase or the entire surgical procedure (Table 2).

During induction, BIS values showed no statistically significant differences between groups (eFigure 2 in Supplement 2). Similarly, no significant differences were detected between the groups in terms of median (IQR) time to extubation (35 min [25-45] vs 30 min [20-40]; *P* = .13), length of PACU stay (75 min [65-85] vs 70 min [62-80]; *P* = .59), and length of hospital stay (8.0 days [7.0-9.0] vs 9.0 days [9.0-10.0]; *P* = .35) or the percentage of time with BIS value between 40 and 60 (73.5% [54.3%-82.2%] vs 63.0% [44.7%-76.0%]; *P* = .06). The QoR-15 scores in the cipepofol and propofol groups were 125.7 (9.4) and 122.2 (12.2), respectively, and the difference was not significant (*P* = .08) (Table 2).

### Safety Outcomes

A total of 99 patients (81.1%) experienced at least 1 adverse event, with significantly fewer adverse events in the cipepofol group (45 [73.8%]) compared to the propofol group (54 [88.5%]; *P* = .04). The most common adverse events, intraoperative hypotension and injection site pain, were less frequent with cipepofol (hypotension: 45 [73.8%] vs 54 [88.5%]; *P* = .04; pain: 6 [9.8%] vs 34 [55.7%]; *P* < .001). Incidences of other adverse events, including postoperative bradycardia, nausea, asthenia, hypertension, tachycardia, and dizziness, were similar between groups (eTable 1 in Supplement 2).

## Discussion

This randomized clinical trial demonstrated that compared with propofol, cipepofol at an equipotent dose provided superior hemodynamic stability during anesthesia induction in patients undergoing TAVR, especially for older patients with severe aortic valve stenosis. Specifically, patients managed with cipepofol significantly reduced the AUC within the first 15 minutes postinduction. The benefit of cipepofol did not seem to be due to compromised anesthesia depth, as there were no differences in the BIS index between the 2 groups within 15 minutes postinduction.

Several studies have shown that cipepofol is both effective and safe for general anesthesia in older patients.^17,27^ Patients who undergo TAVR present with a unique pathophysiology, as postinduction hemodynamic instability occurs more frequently.^9^ To our knowledge, this study was the first to evaluate the use of cipepofol for inducing anesthesia in patients undergoing TAVR, providing important insights into its use in this population.

This study offers more comprehensive insight into the role of cipepofol vs propofol in blood pressure management via a systematic evaluation of the area under the relative MAP curve. Unlike studies that focused solely on transient blood pressure fluctuations, this study examined the effects of cipepofol on blood pressure management throughout the entire induction period, using the AUC as a comprehensive indicator of hemodynamic instability.^26^ The cipepofol group showed a 35% reduction in AUC compared to the propofol group, with a lower incidence of hypotension (70.5% vs 88.5%). The lowest mean (SD) MAPs were 66 (10) mm Hg for cipepofol and 62 (11) mm Hg for propofol. Incidences of postinduction hypertension (8.2% vs 14.8%) and tachycardia (11.5% vs 18.0%) were similar across groups. These findings have clinical relevance, especially for older patients with aortic valve stenosis.

The enhanced hemodynamic stability observed in the cipepofol group is unlikely to be due to more frequent or timely administration of rescue medications. In fact, during anesthesia induction, the total dose of norepinephrine used was 40% lower in the cipepofol group compared to the propofol group (Table 2). Lu and colleagues^28^ reported that in patients aged 75 years and older who underwent hip fracture surgery, the use of epinephrine was lower in the cipepofol group, which is consistent with the findings of the present study. Taken together, these findings imply that compared with propofol, cipepofol may better maintain blood pressure through more direct mechanisms than the use of subsequent pharmacological interventions.

The better postinduction hemodynamic stability observed with cipepofol administration was also not associated with compromised anesthesia depth. Continuous BIS monitoring allowed dynamic assessment of anesthesia depth in both groups, revealing comparable BIS values within 15 minutes postinduction. Thus, the reduced incidence and severity of postinduction hypotension with cipepofol likely did not result from lighter anesthesia.

Previous studies have indicated cipepofol effectively induces general anesthesia in older patients undergoing major noncardiac surgery without serious adverse events.^17,29^ Here, induction with cipepofol, 0.2 mg/kg, and propofol, 1.0 mg/kg, achieved 100% success in both groups. Top-up doses were needed in 7 patients in the cipepofol group (11.5%) and in 9 patients in the propofol group (14.8%), with LOC achieved in less than 1 minute, supporting the efficacy and safety of cipepofol, 0.2 mg/kg, for older patients with cardiovascular compromise.

Our observations may be extrapolated beyond patients undergoing TAVR, as the effects of cipepofol or propofol as an induction agent were assessed within the first 15 minutes after induction but before the surgical intervention began. Therefore, the benefit of cipepofol as an induction agent should be reproducible in patients with severe AS, regardless of whether TAVR is the operation for which anesthesia is being administered.

### Limitations

This study had several limitations. First, as a single-center study, the results may be influenced by the specific patient population, surgical protocols, and care standards of the institution. Multicenter randomized clinical trials are needed to confirm the broader applicability of cipepofol in varied clinical settings. Second, the study did not use blinding for the anesthesia clinicians, which may have introduced some bias in the management of hypotension, particularly regarding vasopressors. Additionally, the study population primarily consisted of high-risk older patients with severe AS. While cipepofol showed benefits over propofol in this hemodynamically vulnerable group, these findings may not extend to other types of surgeries or patients at lower risk. Future studies should include routine cardiac surgeries, noncardiac procedures, and diverse patient populations. Furthermore, this study did not assess the effects of cipepofol throughout the entire intraoperative period or on long-term postoperative outcomes, such as cognitive function and complications. Evaluating these outcomes is essential to fully understand cipepofol’s clinical value, and future studies should consider extended follow-up to assess its long-term safety and efficacy.

## Conclusions

This randomized clinical trial demonstrated that compared with propofol, cipepofol provided superior hemodynamic stability in patients with severe AS during anesthesia induction and may be a safer alternative for this population. Further large-scale trials are needed to determine whether these postinduction benefits translate into better patient outcomes.

## Notes

Supplement 1. Trial Protocol

Supplement 2. eTable 1. Summary of Adverse Events eTable 2. Per-Protocol Analysis for the Outcomes eTable 3. Quality of Recovery-15 (QoR-15) Scale eTable 4. Severity Grading for Pain During Injection and Induction eFigure 1. Comparison of Accumulated Norepinephrine Dose Within 15 Minutes After Anesthesia Induction Between Cipepofol and Propofol eFigure 2. Values in the BIS Within 15 Minutes Following the iInduction of General Anesthesia with Cipepofol versus Propofol eMethods 1. Standardized Surgical Procedure eMethods 2. Statistical Methods

Supplement 3. Data Sharing Statement
