# Supplementary material for: Hemodynamic Impact of Cipepofol vs Propofol During Anesthesia Induction in Patients With Severe Aortic Stenosis: A Randomized Clinical Trial
Source: JAMA Surg. 2025 May 21;160(7):763–70. doi: 10.1001/jamasurg.2025.1299 (PMC12096327; doi:10.1001/jamasurg.2025.1299)
Supplement: Supplement 1. — Trial Protocol [file jamasurg-e251299-s001.pdf]

Study Protocol

Effects of Cipepofol versus Propofol on Hemodynamics after Induction in Aortic Stenosis Patients

A Randomized Clinical Trial

Introduction

Aortic stenosis (AS) is a common type of valvular heart illness that is linked to a high mortality rate among older adults [1]. Its prevalence among individuals aged 65 and above ranges from 2% to 7% [2]. Etiologically, calcification of the valves is the leading cause of AS [3]. This medical condition has a mortality rate of 50% within two years after the commencement of initial signs like angina, syncope, or heart failure [4]. Transcatheter aortic valve replacement (TAVR) has become a widely used, minimally invasive option for treating severe aortic stenosis (AS), as demonstrated by prior research [5]. TAVR has proven efficacy in reducing mortality and enhancing quality of life and functional status, especially for individuals with the highest surgical risk or advanced age or multiple comorbidities. Recently, the scope of treatment has expanded to include individuals who are classified as low- and intermediate-risk [6,7].

TAVR poses a challenge to anesthesiologists because many patients have a severe preexisting condition, making them high-risk [8]. According to prior reports, hypotension is a common side effect of general anesthesia induction, occurring in 53% of noncardiac surgeries [9]. For patients undergoing TAVR with declined physical and physiological function, careful anesthesia management is required to prevent hypotension after general anesthesia induction [10]. Furthermore, a brief drop in blood pressure can result in tissue hypoperfusion and its complications, leading to an elevated risk of postoperative morbidity and mortality [9,11–13]. Research has established that general anesthesia induction-related hypotension (GAIH) is a distinct factor in predicting adverse clinical results [11].

It has been determined that age, ASA physical status, preoperative hypertension, type II diabetes mellitus, and the utilization of propofol for induction are independent predictors of GAIH [14,15]. Propofol, commonly used for induction, has several drawbacks, including dilated blood vessels, decreased cardiac output, and a higher risk of hypotension in individuals over 50 [16,17]. The effects of propofol are more pronounced in older people, even with a reduced dosage of 1-1.5mg kg^{-1} [18]. Cipepofol, an analog of propofol [19], offers a quick onset of effects, shortens the recovery time, reduces injection discomfort, and maintains stable cardiorespiratory function [20]. This makes it a potential substitute for TAVR. Consequently, it is expected that it will reduce the risk of GAIH.

This study aims to explore the effectiveness and safety of cipepofol in induction and maintenance general anesthesia in patients undergoing TAVR to establish its potential for use in clinical practice.

Methods: participants, interventions and outcomes

Study setting

This study will be conducted at the Second Affiliated Hospital Zhejiang University School of Medicine in China.

Eligibility criteria

Inclusion criteria

To be eligible for this study, patients must have met all of the following criteria:

1. Scheduled for elective TAVR;

2. Expected surgery duration of ≥1 to ≤3 hours;
3. Planned use of general anesthesia with endotracheal intubation;
4. Age between 60 and 85 years, with no sex restrictions;
5. Body mass index (BMI) of 18 to 30 kg m^{-2} at screening;
6. American Society of Anesthesiologists (ASA) physical status III–IV;
7. Capable of understanding the study protocol and procedures, willingness to comply with all study requirements, and voluntary provision of written informed consent.

Exclusion Criteria

Patients that meet any of the following criteria will be excluded from this study:

1. Preoperative tracheal intubation;
2. Known allergies to eggs, soy products, opioids, and their reversal agents, or propofol, or contraindications to the use of propofol, opioids, or their reversal agents;
3. Presence of shock or hypotension unresponsive to vasopressors at screening;
4. History of neurological or psychiatric disorders, including traumatic brain injury, seizure, epilepsy, intracranial hypertension, cerebral aneurysm, stroke, schizophrenia, manic disorders or cognitive impairment, or long-term use of psychiatric medications;
5. Hemoglobin (HB) <10.0 g d^{-1} (100 g L^{-1}) at screening;
6. Long-term use of analgesics or sedatives;

Withdrawal Criteria

Patients will be withdrawn from the study if they express an unwillingness to participate before anesthesia induction.

Interventions

Before surgery, all patients will be required to fast for 8 hours. Once they enter the operating room, electrocardiogram (ECG), noninvasive blood pressure (NIBP), cerebral oxygen saturation (ScO_2), peripheral oxygen saturation (SpO_2), and bispectral index (BIS) will be monitored. Two external defibrillator pads (Covidien adult defibrillation electrodes; Medtronic) will be positioned. Before the start, it is necessary to secure arteriovenous and central venous access catheters. Radial artery catheters will be inserted under local infiltration anesthesia with lidocaine, and the arterial sensor will continuously monitor arterial blood pressure. Because patients receiving a CoreValve have a higher risk of heart block, a temporary pacemaker is usually inserted through the internal jugular vein. After 3 minutes of preoxygenation, oxygen will be administered to the patient through a face mask at 6 L/min. Then, 0.2 mg/kg cipepofol or 1 mg/kg propofol will be administered for 30 s until the patient loses consciousness. When the eyelash reflex disappears and the BIS value is ≤60, 20–25 μg/kg alfentanil and 0.6 mg/kg rocuronium will be initiated. Intubation of the trachea shall be accomplished two minutes after the administration of rocuronium. Following that, a ventilator will provide mechanical ventilation, delivering a tidal volume of 6–8 mL/kg at a rate of 12–20 breaths/min, with a 1:2 inhalation-to-exhalation ratio, an oxygen flow

of 2 L/min, and maintaining an end-expiratory PETCO2 level of 35–45 mmHg. Our DoCare system recorded mean arterial pressure (MAP) with a 30 s sampling period during the anesthetic procedure. The baseline MAP will be defined as the mean MAP during the 5-minute preceding induction.

At first, cipepofol is given at a speed of 0.8 mg $kg^{-1}h^{-1}$, and the dosage is adjusted as needed (ranging from 0.25 to 0.5 mg $kg^{-1}h^{-1}$ per time), with the maximum infusion rate being 1.5 mg $kg^{-1}h^{-1}$. Propofol is given initially at 4.0 mg $kg^{-1}h^{-1}$ adjusted as appropriate (1-2 mg $kg^{-1}h^{-1}$ per time), with maximum allowed infusion rates of 7.0 mg $kg^{-1}h^{-1}$. Alfentanil will be infused at a rate of 20–60 μg $kg^{-1}h^{-1}$, with additional doses of 5–10 μg/kg allowed if needed. The administration of the experimental drug will be halted when the surgical procedures end. Rocuronium will be intermittently injected on an as-needed basis. In patients who did not experience complications during the surgery, sugammadex (IV, 2 mg/kg) or atropine (IV, 0.02 mg/kg) plus neostigmine (IV, 0.03 mg/kg) will then be administrated to reverse residual muscle relaxation at the end of surgery. The amount of crystalloids and colloids to be administered (including blood) will be determined by the preoperative hematocrit, intraoperative hemodynamics, and the quantity of blood lost. The patients will receive heparin during the operation to keep their activated clotting time at a minimum of 250 seconds, and protamine will be administered when the operation is finished to reverse the effects of heparin.

The INVOS 5100c surface pads (Covidien, Mansfield, MA) will be applied bilaterally on the forehead in order to monitor the ScO_2 continuously, and the resultant values will undergo statistical analysis. The baseline values will be

obtained before anesthesia induction and oxygen administration. The drug doses will be modified based on MAP, HR, and BIS values to keep the BIS value within the range of 40 to 60. To monitor cardiac index (CI), systemic vascular resistance index (SVRI), and stroke volume variation (SVV), the PULSION Medical Systems (Gothenburg, Vastra Gotaland, Sweden) will be utilized. Hemodynamic measurements will be taken at various points during the procedure, including the start, after induction, during rapid ventricular pacing, after the valve has been implanted, and when the process is finished.

Hypotension is diagnosed when the MAP is less than 65 mmHg or decreases by more than 20% from the initial value at the time of induction. A single intravenous injection of norepinephrine bolus of 5 μg is administered to treat intraoperative hypotension. A continuous norepinephrine infusion will be initiated if two or more bolus administrations are required. For heart rates below 50 beats/min, 0.5 mg of atropine is to be given.

Outcomes

Primary and Secondary Outcomes

The primary outcome measure will be the AUC during the initial 15 minutes postinduction. Secondary outcomes will include the incidence of hypotension, need for temporary pacemaker activation (HR < 50 bpm), lowest MAP, cumulative vasopressor dose, anesthetic dosage, BIS index within 15 minutes postinduction, time to LOC, percentage of time with a BIS value of 40 – 60, and postoperative recovery indicators (extubation time, postanesthesia care unit (PACU) stay duration, hospital stay length, and Quality of Recovery-15 (QoR-15) score).

Safety Outcomes

Safety will be evaluated in terms of the frequency of adverse events, alterations in vital signs, postoperative recovery, and injection-site pain, which will be assessed on a four-point scale.

Sample size

The primary objective of this experiment is to measure the area of the MAP baseline curve within 15 min of general anesthesia induction. Previous clinical studies demonstrate that propofol's mean (standard deviation) is –10706.429 (6877.0052). Anesthesia experts agreed that a 33% difference in MAP baseline curve area between the two groups within 15 min of induction is clinically relevant. This must be statistically significant at a confidence level of 0.05 with an 80% confidence interval. To account for a 5% dropout rate, 124 subjects are required, with 62 in the group receiving a propofol injection and 62 in the group receiving a cipepofol injection.

Recruitment

Recruitment for the study will be carried out among the inpatients of the Second Affiliated Hospital Zhejiang University School of Medicine, beginning in June 2023 and concluding in June 2024.

Assignment of interventions: allocation

Sequence generation

An independent investigator will use a computer-generated random number sequence to allocate qualified participants to either the test drug group (cipepofol) or the control group (propofol) in a 1:1 ratio.

Concealment mechanism

The allocation information will be kept secret through numbered, opaque-sealed envelopes. The envelopes will be stored securely throughout the study and opened by the researchers when the participant arrives in the operating room.

Implementation

The attending anesthesiologists will receive sequentially numbered opaque envelopes on the day of the surgery, which have been generated per the randomization schedule.

Assignment of interventions: blinding

To maintain the integrity of this single-blind study, only the patients will be kept unaware of their treatment details. Given the need to adjust the doses of propofol and cipepofol during the operation, the attending anesthesiologists will not be blinded during drug administration to ensure patient safety. However, the outcome assessors and data analysts will remain blinded to group assignments to minimize potential bias. Additionally, researchers involved in drug allocation, preparation, and administration will not participate in outcome evaluation to further safeguard objectivity.

Data collection and management

The MAP will be continuously recorded and averaged every 30 seconds by a DoCare information system. An in-room observer will record the number of boluses and the total amount of vasoactive drugs administered in the first 15 minutes after anesthesia induction. Other data describing patient characteristics, surgical procedures, and anesthetics will be extracted from the electronic anesthesia records or clinical notes of the patient.

Baseline features of patients

Following approval, a researcher working autonomously will gather initial data the day before the operation. All personal information will remain private and will only be used for research. Demographic data will be gathered the day prior to surgery, including gender, age, vital signs, BMI, medical history, family history, smoking and drinking habits, allergies, prior operations and anesthesia, and medications. Additionally, ASA classification, Society of Thoracic Surgeons (STS) risk score, and New York Heart Association (NYHA) Functional Classification will be assessed. Tests such as ECG, cardiac computed tomography angiography (CTA), echocardiography, and diffusion-weighted magnetic resonance imaging (DW-MRI) will also be conducted. Lastly, the most recent laboratory values, including hemoglobin (HB), creatinine, serum albumin, blood urea nitrogen (BUN), CK-MB, cardiac troponin T (cTnT), N-terminal pro-B-type natriuretic peptide (NT-pro-BNP), and interleukin 6 (IL-6) will be evaluated.

Assessment of the patient during and after anesthesia.

During anesthesia, the following will be monitored: vital sign parameters, total dose or infusion rate of anesthetic agents, vasoactive drugs, ScO_2, BIS, CI, SVRI, estimated

blood loss, urine volume, type and dose of fluids and blood transfusions, duration of surgery and anesthesia, valve type, pre- and post-dilatation times, and contrast dose. The postoperative laboratory, imaging, and electrocardiogram examination results will be preserved. QoR-15 will also be recorded.

Data management

A paper-based questionnaire will be used to collect data, which will remain anonymous. The Second Affiliated Hospital Zhejiang University School of Medicine will securely store the information recorded on paper forms in accordance with data protection procedures. To avoid data entry mistakes, a fixed dual researcher will enter the data and manually review it. The data will be saved in Microsoft Excel spreadsheets.

Confidentiality

For the purpose of preserving personal information and contacts, a unique alpha-numeric ID will be given to each patient in order to guarantee privacy.

Statistical methods

This study will adhere to the intention-to-treat principle for the full analysis set, including all patients who will be randomly assigned to either the cipepofol or propofol group. For patients who will require conversion to open-chest surgery, only data collected before the conversion will be included to preserve the integrity of randomization and minimize selection bias.

For the primary outcome measure, the Wilcoxon rank-sum test will be used to compare the AUC within the first 15 minutes after anesthesia induction between groups. The AUC below the baseline MAP will be calculated as follows:

$\sum(((S_i-S_{baseline})+(S_{i-1}-S_{baseline}))/2\times\Delta X)$

where $S_{baseline}$ is the baseline MAP, S_i is the MAP at minute i (i=1,…,15), and ΔX is the time interval between measurements.

We will use the log-rank test to compare group differences in the time from drug discontinuation to extubation, postanesthesia care unit (PACU) stay, and hospital stay, accounting for censored data through appropriate survival analysis methods.

Data distributions will be assessed using the Shapiro–Wilk test. Continuous variables will be expressed as the means ± standard deviations (SDs) if normally distributed or medians (IQRs) if not normally distributed, and comparisons between groups will be made with Student's t test or the Mann–Whitney U test, respectively. Binary variables will be presented as frequencies and percentages, and differences between groups will be compared using the chi-square test or Fisher's exact test. For all tests, statistical significance will be set at $P < 0.05$. Statistical analyses will be conducted in SAS Enterprise Guide 8.3, and visualizations will be created with GraphPad Prism 9.1.0.

Oversight and monitoring

Composition of the coordinating center and trial steering committee

The study research team will be responsible for conducting the trial. The principal investigator is in charge of supervising the trial and making sure that it adheres to the study protocol.

Composition of the data monitoring committee, its role, and reporting structure

The principal investigator will conduct regular reviews of the data and trial progress every three weeks; this study has no data monitoring committee.

Adverse event reporting and harms

Assessing safety during anesthesia will involve tracking vital signs and noting any adverse events (AEs) or serious adverse events (SAEs). Any undesirable medical event for a patient, regardless of its connection to the research protocol, is referred to as an adverse event.

Frequency and plans for auditing trial conduct

Annually, the research office and ethics committee of the department will audit the study, while the principal investigator will assess the accuracy of the data and the trial's progression every three weeks.

Plans for communicating important protocol amendments to relevant parties (e.g., trail participants, ethical committees)

The principal investigator is in charge of any protocol modifications and their implementation. The necessary modifications must be approved by the ethics committee before they can be applied. The principal investigator is responsible for communicating the changes to the protocol and ensuring that all study personnel are properly trained in regard to the amendments.

Dissemination plans

All participants will be required to give written consent before any protocol-specified procedures or assessments are conducted. The findings of the study will be shared at relevant conferences and published in international peer-reviewed journals.

References

1. Guinot P-G, Depoix J-P, Etchegoyen L, Benbara A, Provenchère S, et al. Anesthesia and Perioperative Management of Patients Undergoing Transcatheter Aortic Valve Implantation: Analysis of 90 Consecutive Patients With Focus on Perioperative Complications. J Cardiothorac Vasc Anesth. 2010;24:752–61.

2. Martinsson A, Li X, Andersson C, Nilsson J, Smith JG, et al. Temporal Trends in the Incidence and Prognosis of Aortic Stenosis. Circulation. 2015;131:988–94.

3. Members WG, Roger VL, Go AS, Lloyd-Jones DM, Benjamin EJ, et al. Heart Disease and Stroke Statistics—2012 Update. Circulation. 2012;125:e2–220.

4. Rex S. Anesthesia for transcatheter aortic valve implantation: an update. Current Opinion in Anaesthesiology. 2013;26:456–66.

5. Ponikowski P, Voors AA, Anker SD, Bueno H, Cleland JGF, et al. 2016 ESC Guidelines for the Diagnosis and Treatment of Acute and Chronic Heart Failure. Rev Esp Cardiol (Engl Ed). 2016;69:1167.

6. Gargiulo G, Sannino A, Capodanno D, Barbanti M, Buccheri S, et al. Transcatheter Aortic Valve Implantation Versus Surgical Aortic Valve Replacement. Ann Intern Med. 2016;165:334.

7. Siontis GCM, Praz F, Pilgrim T, Mavridis D, Verma S, et al. Transcatheter aortic valve implantation vs. surgical aortic valve replacement for treatment of severe aortic stenosis: a meta-analysis of randomized trials. Eur Heart J. 2016;37:3503–12.

8. Klein AA, Skubas NJ, Ender J. Controversies and Complications in the Perioperative Management of Transcatheter Aortic Valve Replacement. Anesthesia Analg. 2014;119:784–98.

9. Maheshwari K, Turan A, Mao G, Yang D, Niazi AK, et al. The association of hypotension during non-cardiac surgery, before and after skin incision, with postoperative acute kidney injury: a retrospective cohort analysis. Anaesthesia. 2018;73:1223–8.

10. Südfeld S, Brechnitz S, Wagner JY, Reese PC, Pinnschmidt HO, et al. Post-induction hypotension and early intraoperative hypotension associated with general anaesthesia. Br J Anaesth. 2017;119:57–64.

11. Green RS, Butler MB. Postintubation Hypotension in General Anesthesia. J Intensive Care Med. 2016;31:667–75.

12. Sun LY, Wijeysundera DN, Tait GA, Scott Beattie W. Association of Intraoperative Hypotension With Acute Kidney Injury After Elective Noncardiac Surgery. Surv Anesthesiol. 2016;60:120–1.

13. Bijker JB, Persoon S, Peelen LM, Moons KGM, Kalkman CJ, et al. Intraoperative Hypotension and Perioperative Ischemic Stroke after General Surgery. Anesthesiology. 2012;116:658–64.

14. Kawasaki S, Kiyohara C, Tokunaga S, Hoka S. Prediction of hemodynamic fluctuations after induction of general anesthesia using propofol in non-cardiac surgery: a retrospective cohort study. BMC Anesthesiol. 2018;18:167.

15. Reich DL, Hossain S, Krol M, Baez B, Patel P, et al. Predictors of Hypotension After Induction of General Anesthesia. Anesthesia Analg. 2005;101:622–8.

16. Akhtar S, Liu J, Heng J, Dai F, Schonberger RB, et al. Does intravenous induction dosing among patients undergoing gastrointestinal surgical procedures follow current recommendations: a study of contemporary practice. J Clin Anesth. 2016;33:208–15.

17. Carlier S, Aken HV, Vandermeersch E, Thorniley A, Byttebier G. Does Nitrous Oxide Affect the Hemodynamic Effects of Anesthesia Induction with Propofol? Anesthesia Analg. 1989;68:728-33.

18. Jor O, Maca J, Koutna J, Gemrotova M, Vymazal T, et al. Hypotension after induction of general anesthesia: occurrence, risk factors, and therapy. A prospective multicentre observational study. J Anesth. 2018;32:673–80.

19. Bian Y, Zhang H, Ma S, Jiao Y, Yan P, et al. Mass balance, pharmacokinetics and pharmacodynamics of intravenous HSK3486, a novel anaesthetic, administered to healthy subjects. Br J Clin Pharmacol. 2020;87:93–105.

20. Teng Y, Ou M, Wang X, Zhang W, Liu X, et al. Efficacy and safety of ciprofol for the sedation/anesthesia in patients undergoing colonoscopy: Phase IIa and IIb multi-center clinical trials. Eur J Pharm Sci. 2021;164:105904.
