# Supplementary material for: Hemodynamic Impact of Cipepofol vs Propofol During Anesthesia Induction in Patients With Severe Aortic Stenosis: A Randomized Clinical Trial
Source: JAMA Surg. 2025 May 21;160(7):763–70. doi: 10.1001/jamasurg.2025.1299 (PMC12096327; doi:10.1001/jamasurg.2025.1299)
Supplement: Supplement 2. — eTable 1. Summary of Adverse Events eTable 2. Per-Protocol Analysis for the Outcomes eTable 3. Quality of Recovery-15 (QoR-15) Scale eTable 4. Severity Grading for Pain During Injection and Induction eFigure 1. Comparison of Accumulated Norepinephrine Dose Within 15 Minutes After Anesthesia Induction Between Cipepofol and Propofol eFigure 2. Values in the BIS Within 15 Minutes Following the iInduction of General Anesthesia with Cipepofol versus Propofol eMethods 1. Standardized Surgical Procedure eMethods 2. Statistical Methods [file jamasurg-e251299-s002.pdf]

Supplemental Online Content

Ni T, Zhou X, Wu S, et al. Hemodynamic impact of cipepofol vs propofol during anesthesia induction in patients with severe aortic stenosis: a randomized clinical trial. *JAMA Surg*. Published online May 21, 2025. doi:10.1001/jamasurg.2025.1299

eTable 1. Summary of Adverse Events

eTable 2. Per-Protocol Analysis for the Outcomes

eTable 3. Quality of Recovery-15 (QoR-15) Scale

eTable 4. Severity Grading for Pain During Injection and Induction

eFigure 1. Comparison of Accumulated Norepinephrine Dose Within 15 Minutes After Anesthesia Induction Between Cipepofol and Propofol

eFigure 2. Values in the BIS Within 15 Minutes Following the iInduction of General Anesthesia with Cipepofol versus Propofol

eMethods 1. Standardized Surgical Procedure

eMethods 2. Statistical Methods

This supplemental material has been provided by the authors to give readers additional information about their work.

© 2025 Ni T et al. *JAMA Surgery*.

eTable 1. Summary of Adverse Events

**Category**	**Cipepofol (n = 61) , No. (%)**	**Propofol (n = 61) , No. (%)**	**Total (n = 122) , No. (%)**	***P* value**
Hypotension	45 (73.8)	54 (88.5)	99 (81.1)	.04
Bradycardia	6 (9.8)	10 (16.4)	16 (13.1)	.28
Injection-site pain	6 (9.8)	34 (55.7)	40 (32.8)	< .001
Nausea	3 (4.9)	5 (8.2)	8 (6.6)	.72
Asthenia	2 (3.3)	5 (8.2)	7 (5.7)	.44
Vomiting	0	1 (1.6)	1 (0.8)	>.99
Hypertension	5 (8.2)	9 (14.8)	14 (11.5)	.26
Tachycardia	7 (11.5)	11 (18)	18 (14.8)	.31
Dizziness	2 (3.3)	1 (1.6)	3 (2.5)	>.99

© 2025 Ni T et al. *JAMA Surgery*.

eTable 2. Per-Protocol Analysis for the Outcomes

**Variables**	**Cipepofol (n = 61)**	**Propofol (n = 60)**	**P value**
Primary outcome, median (IQR), mmHg·s			
AUC below the baseline MAP	−8505.0 (−12402.8- −5184.0)	−13330.7 (−17041.7- −8174.5)	< .001
Secondary outcomes			
Post-induction hypotension, No. (%)	43 (70.5)	53 (88.3)	.02
Lowest MAP, mean (SD), mmHg	66.4 (9.5)	61.4 (10.7)	.007
Pacemaker activation, No. (%)	5 (8.2)	8 (13.3)	.36
Vasopressor use, median (IQR), µg			
Norepinephrine dose within first 15 minutes of induction	6.0 (0.0–10.0)	10.0 (5.0–20.0)	.007
Norepinephrine dose during the entire procedure	356.0 (156.0–553.0)	448.5 (287.3-729.0)	.03
Epinephrine dose during the entire procedure	14.0 (0.0–67.0)	20.0 (0.0–52.0)	.75
Time to LOC, median (IQR), s	56 (50–63)	55 (49–61)	.58
Dose of cipepofol/propofol, median (IQR), mg			
For induction	12.0 (10.0–14.2)	60.0 (50.0–70.0)	< .001
Total amount of drug administered	120.0 (102.5–152.5)	539.0 (424.0–615.0)	< .001
Top-up dose during induction, No. (%)	7 (11.5)	9 (15)	.57
Dose of alfentanil, median (IQR), µg			
For induction	1200.0 (1000.0–1400.0)	1200.0 (1000.0–1400.0)	.98
Total amount of drug administered	2800.0 (2520.0–3200.0)	2725.0 (2325.0–3080.0)	.34
Dose of rocuronium, median (IQR), mg			
For induction	35.0 (30.0–42.0)	36.5 (32.8–41.0)	.55
Total amount of drug administered	39.0 (34.0–45.0)	40.0 (34.0–47.0)	.54

Abbreviations: AUC: area under the curve; BIS: bispectral index; LOC: loss of consciousness; MAP: mean arterial pressure; PACU: post-anesthesia care unit; PIH: post-induction hypotension; QoR-15: Quality of Recovery-15; SD: standard deviation; IQR: interquartile range.

© 2025 Ni T et al. *JAMA Surgery*.

eTable 3. Quality of Recovery-15 (QoR-15) Scale

How Have You Been Feeling in the Last 24 Hours?

1. Able to breathe easily	None of the time	0	1	2	3	4	5	6	7	8	9	10	All of the time
2. Able to enjoy food	None of the time	0	1	2	3	4	5	6	7	8	9	10	All of the time
3. Able to feel rested	None of the time	0	1	2	3	4	5	6	7	8	9	10	All of the time
4. Able to sleep well	None of the time	0	1	2	3	4	5	6	7	8	9	10	All of the time
5. Able to look after personal hygiene unaided	None of the time	0	1	2	3	4	5	6	7	8	9	10	All of the time
6. Able to communicate with family or friends	None of the time	0	1	2	3	4	5	6	7	8	9	10	All of the time
7. Able to get support from the hospital doctors and nurses	None of the time	0	1	2	3	4	5	6	7	8	9	10	All of the time
8. Able to return to work or usual home activities	None of the time	0	1	2	3	4	5	6	7	8	9	10	All of the time
9. Able to comfortable and in control	None of the time	0	1	2	3	4	5	6	7	8	9	10	All of the time
10. Have a feeling of general well-being	None of the time	0	1	2	3	4	5	6	7	8	9	10	All of the time

Have You Had Any of the Following in the Last 24 Hours?

1. Moderate pain	None of the time	10	9	8	7	6	5	4	3	2	1	0	All of the time
2. Severe pain	None of the time	10	9	8	7	6	5	4	3	2	1	0	All of the time
3. Nausea or vomiting	None of the time	10	9	8	7	6	5	4	3	2	1	0	All of the time
4. Worry or anxiety	None of the time	10	9	8	7	6	5	4	3	2	1	0	All of the time
5. Sadness or depression	None of the time	10	9	8	7	6	5	4	3	2	1	0	All of the time

Notes:
10 to 0, where 10 = none of the time [excellent] and 0 = all of the time [poor].

© 2025 Ni T et al. *JAMA Surgery*.

eTable 4. Severity Grading for Pain During Injection and Induction

Grade	Meaning
0	No pain (no reaction at the injection site)
1	Mild pain (slight verbal, facial, or motor response to injection pain)
2	Moderate pain (clear verbal, facial, or motor response to injection pain)
3	Severe pain (complaints of pain and arm withdrawal)

© 2025 Ni T et al. *JAMA Surgery*.

eFigure 1. Comparison of Accumulated Norepinephrine Dose Within 15 Minutes After Anesthesia Induction Between Cipepofol and Propofol

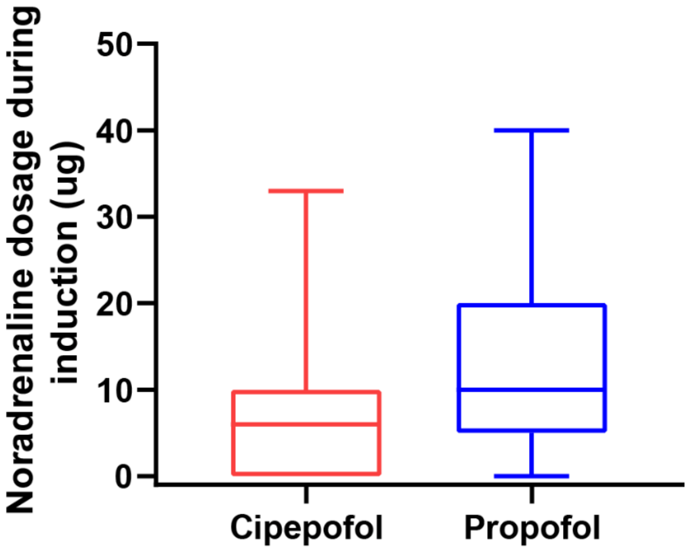

This figure presents the accumulated norepinephrine dosage administered within the first 15 minutes after the induction of general anesthesia in both groups. The median dosage was significantly lower in the cipepofol group (6.0 µg [0.0–10.0]) than in the propofol group (10.0 µg [5.0–20.0]); the difference was statistically significant (*P* = .006).

© 2025 Ni T et al. *JAMA Surgery*.

eFigure 2. Values in the BIS Within 15 Minutes Following the iInduction of General Anesthesia with Cipepofol versus Propofol

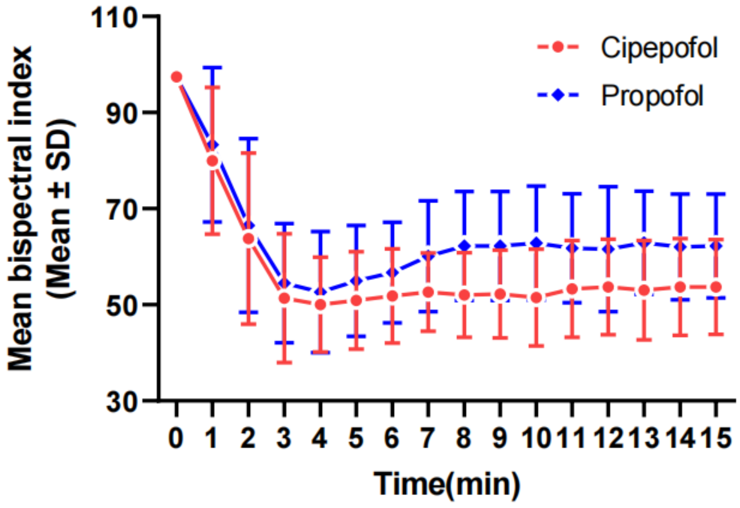

Mean bispectral index (BIS) values over 15 minutes post-anesthesia induction with cipepofol and propofol, showing similar sedation levels across time points (*P* > .05).

© 2025 Ni T et al. *JAMA Surgery*.

eMethods 1. Standardized Surgical Procedure

All patients fasted for 8 hours before the operation. Upon entering the operating room, electrocardiogram (ECG), noninvasive blood pressure (NIBP), cerebral oxygen saturation (ScO_2), peripheral oxygen saturation (S_PO_2), and bispectral index (BIS) monitoring were commenced. External defibrillator pads (Covidien adult electrodes, Medtronic) were applied, and central venous access was established. Radial artery catheters were placed prior to induction under local lidocaine for continuous arterial pressure monitoring. Due to the increased risk of heart block in patients receiving the CoreValve, an artificial valve manufactured by Medtronic and commonly used in TAVR procedures, a temporary pacemaker was typically inserted via the internal jugular vein; other valve types may be used depending on patient-specific anatomical or clinical indications. Preoxygenation was carried out with 6 L/min of oxygen via a face mask. Once the expired oxygen concentration reached 80% or greater, induction was started.

For the propofol group, patients received 1 mg/kg propofol (Fresenius Kabi Deutschland GmbH, Germany) over 30 seconds until loss of consciousness (LOC), defined as the disappearance of the eyelash reflex, occurred. If LOC had not occurred within 1 min after the initial administration of propofol, a top-up dose of 50% of the initial dose was given. Another top-up dose was subsequently administered if LOC had not occurred within 2 min after the initial administration of propofol. Subsequently, 20–25 μg/kg alfentanil and 0.6 mg/kg rocuronium were administered, followed by tracheal intubation 2 minutes later. Volume-controlled ventilation started with a tidal volume of 6–8 mL/kg, 12–20 breaths/min, a 1:2 inhalation-to-exhalation ratio, 2 L/min oxygen flow, and $PETCO_2$ maintained at 35–45 mmHg. The baseline MAP was defined as the

© 2025 Ni T et al. *JAMA Surgery*.

average of the MAP 5 minutes prior to induction. The sedation depth was targeted at a BIS index between 40 and 60 by titrating the propofol infusion rate.

For the cipepofol (produced by Shenyang Haisco Pharmaceutical Co., Ltd., China) group, the patient was inducted with 0.2 mg/kg cipepofol, and the target anesthesia depth was achieved by titrating the cipepofol rate to achieve the same BIS as for the propofol group. The rest of the induction protocol was the same as that used for the propofol group.

Propofol or cipepofol infusion ceased upon completion of the surgery. Residual muscle paralysis was reversed with the administration of either sugammadex or a combination of atropine and neostigmine to ensure that the train-of-four (TOF) ratio was 0.9 or greater.

The volumes of crystalloids, colloids, and blood products were adjusted intraoperatively by the care team based on the patient's preoperative hematocrit, intraoperative hemodynamics, and extent of blood loss during surgery. To maintain an activated clotting time (ACT) of at least 250 seconds, heparin was administered intraoperatively, and its effects were reversed postoperatively with protamine. The endotracheal tube was removed once the patient had regained consciousness and demonstrated spontaneous and stable respiration, stable hemodynamics, and no signs of bleeding. Following extubation, the patient was transferred to the intensive care unit (ICU) for postoperative care. The Quality of Recovery-15 (QoR-15) score was assessed 24 hours postoperatively

Post-induction hypotension (PIH), defined as a MAP reduction of >20% from baseline or a MAP <65 mmHg for at least 1 minute. Any MAP reduction >20% from

© 2025 Ni T et al. *JAMA Surgery*.

baseline or a MAP <65 mmHg for at least 1 minute within the first 15 minutes after general anesthesia induction triggered up to two norepinephrine boluses (5 μg every minute). If hypotension persisted after two boluses, a norepinephrine infusion (0.02–0.3 μg/kg/min) was initiated and continued until the MAP was no longer reduced by >20% or reached ≥ 65 mmHg. If hypotension reoccurred, the same algorithm, starting with two boluses, was repeated. The baseline MAP was defined as the mean MAP during the 5-minute preceding induction.

© 2025 Ni T et al. *JAMA Surgery*.

eMethods 2. Statistical Methods

This study adhered to the intention-to-treat principle for the full analysis set, including all patients randomly assigned to either the cipepofol or propofol group. For patients who required conversion to open-chest surgery, only data collected before the conversion were included to preserve the integrity of randomization and minimize selection bias.

For the primary outcome measure, the Wilcoxon rank-sum test was used to compare the AUC within the first 15 minutes after anesthesia induction between groups. The AUC below the baseline MAP was calculated as:

$\sum(((S_i-S_{baseline})+(S_{i-1}-S_{baseline}))/2\times\Delta X)$

where $S_{baseline}$ is the baseline MAP, S_i is the MAP at minute i (i=1,…,15), and ΔX is the time interval between measurements.

We used the log-rank test to compare group differences in the time from drug discontinuation to extubation, postanesthesia care unit (PACU) stay, and hospital stay, accounting for censored data through appropriate survival analysis methods.

Data distributions were assessed using the Shapiro–Wilk test. Continuous variables are expressed as the means ± standard deviations (SDs) if normally distributed or medians (IQRs) if not normally distributed, and comparisons between groups were made with Student's t test or the Mann–Whitney U test, respectively. Binary variables are presented as frequencies and percentages, and differences between groups are compared using the chi-square test or Fisher's exact test. For all tests, statistical significance was set at $P < .05$. Statistical analyses were conducted in SAS Enterprise Guide 8.3, and visualizations were created with GraphPad Prism 9.1.0.

© 2025 Ni T et al. *JAMA Surgery*.
