# Supplementary material for: Hemodynamic Impact of Cipepofol vs Propofol During Anesthesia Induction in Patients With Severe Aortic Stenosis: A Randomized Clinical Trial
Source: JAMA Surg. 2025 May 21;160(7):763–70. doi: 10.1001/jamasurg.2025.1299 (PMC12096327; doi:10.1001/jamasurg.2025.1299)
Supplement: Supplement 3. — Data Sharing Statement [file jamasurg-e251299-s003.pdf]

Data Sharing Statement

Ni. Hemodynamic Impact of Cipepofol vs Propofol During Anesthesia Induction in Patients With Severe Aortic Stenosis. *JAMA Surg*. Published May 21, 2025.
doi:10.1001/jamasurg.2025.1299

Data
Additional Information: ClinicalTrials.gov Identifier: NCT05881291
https://register.clinicaltrials.gov/ clinicaltrial
Data available: No
